# Supplementary material for: Modeling glioblastoma heterogeneity as a dynamic network of cell states
Source: Mol Syst Biol. 2021 Sep 16;17(9):e10105. doi: 10.15252/msb.202010105 (PMC8444284; doi:10.15252/msb.202010105)
Supplement: Supplementary file 6 — Source Data for Figure 5 [file MSB-17-e10105-s004.zip › Figure5A_sourcedata/GSEA_3017/nosigdb_stateC.GseaPreranked.1622795002785/VANGRONINGEN_MES.html]

Details for gene set VANGRONINGEN\_MES[GSEA]

|  || Dataset | state13017 |
| Phenotype | NoPhenotypeAvailable |
| Upregulated in class | na\_neg |
| GeneSet | VANGRONINGEN\_MES |
| Enrichment Score (ES) | -0.4742375 |
| Normalized Enrichment Score (NES) | -2.8968546 |
| Nominal p-value | 0.0 |
| FDR q-value | 0.0 |
| FWER p-Value | 0.0 |
Table: GSEA Results Summary

  

Fig 1: Enrichment plot: VANGRONINGEN\_MES      
 Profile of the Running ES Score & Positions of GeneSet Members on the Rank Ordered List

  

| PROBE | GENE SYMBOL | GENE\_TITLE | RANK IN GENE LIST | RANK METRIC SCORE | RUNNING ES | CORE ENRICHMENT || 1 | DLX1 |  |  | 32 | 0.469 | -0.0419 | No |
| 2 | GAS1 |  |  | 64 | 0.384 | -0.0852 | No |
| 3 | GALNT10 |  |  | 197 | -0.251 | -0.3236 | No |
| 4 | ARL4A |  |  | 224 | -0.259 | -0.3624 | No |
| 5 | KDM5B |  |  | 230 | -0.260 | -0.3617 | No |
| 6 | PPIB |  |  | 265 | -0.268 | -0.4152 | No |
| 7 | EHD2 |  |  | 275 | -0.270 | -0.4216 | No |
| 8 | RRBP1 |  |  | 283 | -0.271 | -0.4242 | No |
| 9 | L3HYPDH |  |  | 298 | -0.276 | -0.4398 | No |
| 10 | LIFR |  |  | 311 | -0.282 | -0.4514 | No |
| 11 | PLEKHH2 |  |  | 319 | -0.284 | -0.4535 | No |
| 12 | CRISPLD1 |  |  | 331 | -0.288 | -0.4630 | No |
| 13 | ARMCX2 |  |  | 338 | -0.290 | -0.4630 | Yes |
| 14 | MMP2 |  |  | 340 | -0.291 | -0.4535 | Yes |
| 15 | DLC1 |  |  | 347 | -0.296 | -0.4533 | Yes |
| 16 | MEST |  |  | 352 | -0.299 | -0.4492 | Yes |
| 17 | COL4A2 |  |  | 353 | -0.300 | -0.4375 | Yes |
| 18 | UGDH |  |  | 360 | -0.301 | -0.4371 | Yes |
| 19 | SPRY1 |  |  | 369 | -0.307 | -0.4402 | Yes |
| 20 | LMNA |  |  | 376 | -0.308 | -0.4394 | Yes |
| 21 | CREB3L2 |  |  | 378 | -0.309 | -0.4293 | Yes |
| 22 | IGFBP5 |  |  | 384 | -0.311 | -0.4266 | Yes |
| 23 | CETN2 |  |  | 392 | -0.317 | -0.4274 | Yes |
| 24 | TIMP1 |  |  | 399 | -0.320 | -0.4263 | Yes |
| 25 | TPM1 |  |  | 407 | -0.327 | -0.4267 | Yes |
| 26 | CALD1 |  |  | 420 | -0.335 | -0.4362 | Yes |
| 27 | ABRACL |  |  | 425 | -0.337 | -0.4306 | Yes |
| 28 | KLF6 |  |  | 429 | -0.339 | -0.4231 | Yes |
| 29 | INSIG1 |  |  | 434 | -0.342 | -0.4173 | Yes |
| 30 | WWTR1 |  |  | 437 | -0.345 | -0.4076 | Yes |
| 31 | WLS |  |  | 438 | -0.345 | -0.3942 | Yes |
| 32 | FAT1 |  |  | 441 | -0.346 | -0.3845 | Yes |
| 33 | RAB31 |  |  | 445 | -0.349 | -0.3766 | Yes |
| 34 | HSPA5 |  |  | 447 | -0.350 | -0.3649 | Yes |
| 35 | MYL12A |  |  | 448 | -0.350 | -0.3512 | Yes |
| 36 | SERPINE2 |  |  | 452 | -0.355 | -0.3431 | Yes |
| 37 | DKK3 |  |  | 454 | -0.356 | -0.3311 | Yes |
| 38 | RHOC |  |  | 455 | -0.359 | -0.3171 | Yes |
| 39 | B2M |  |  | 468 | -0.371 | -0.3253 | Yes |
| 40 | HES1 |  |  | 470 | -0.373 | -0.3126 | Yes |
| 41 | FIBIN |  |  | 478 | -0.379 | -0.3110 | Yes |
| 42 | NQO1 |  |  | 479 | -0.381 | -0.2962 | Yes |
| 43 | RAB13 |  |  | 482 | -0.388 | -0.2849 | Yes |
| 44 | IFITM2 |  |  | 483 | -0.388 | -0.2698 | Yes |
| 45 | VCL |  |  | 484 | -0.388 | -0.2547 | Yes |
| 46 | ATXN1 |  |  | 486 | -0.392 | -0.2414 | Yes |
| 47 | SCRG1 |  |  | 508 | -0.434 | -0.2640 | Yes |
| 48 | NES |  |  | 509 | -0.435 | -0.2470 | Yes |
| 49 | PTN |  |  | 517 | -0.446 | -0.2428 | Yes |
| 50 | CTSC |  |  | 531 | -0.470 | -0.2490 | Yes |
| 51 | IFITM3 |  |  | 533 | -0.477 | -0.2324 | Yes |
| 52 | COL6A1 |  |  | 534 | -0.479 | -0.2137 | Yes |
| 53 | COL4A1 |  |  | 545 | -0.507 | -0.2128 | Yes |
| 54 | TNFRSF12A |  |  | 550 | -0.519 | -0.2001 | Yes |
| 55 | GJA1 |  |  | 552 | -0.527 | -0.1815 | Yes |
| 56 | GDF15 |  |  | 554 | -0.531 | -0.1627 | Yes |
| 57 | SPARCL1 |  |  | 557 | -0.544 | -0.1453 | Yes |
| 58 | ANXA2 |  |  | 563 | -0.567 | -0.1327 | Yes |
| 59 | TNC |  |  | 564 | -0.572 | -0.1104 | Yes |
| 60 | SPARC |  |  | 567 | -0.575 | -0.0918 | Yes |
| 61 | CD44 |  |  | 575 | -0.659 | -0.0794 | Yes |
| 62 | VIM |  |  | 578 | -0.674 | -0.0569 | Yes |
| 63 | TM4SF1 |  |  | 580 | -0.689 | -0.0320 | Yes |
| 64 | CYR61 |  |  | 584 | -0.714 | -0.0099 | Yes |
| 65 | EMP1 |  |  | 589 | -0.785 | 0.0132 | Yes |
Table: GSEA details [plain text format]

  

Fig 2: VANGRONINGEN\_MES: Random ES distribution      
 Gene set null distribution of ES for **VANGRONINGEN\_MES**
